# Effects of Different Proportions of DHA and ARA on Cognitive Development in Infants: A Meta-Analysis

**DOI:** 10.3390/nu17061091

**Published:** 2025-03-20

**Authors:** Ailing Tian, Lirong Xu, Ignatius Man-Yau Szeto, Xuemin Wang, Duo Li

**Affiliations:** 1 Institute of Nutrition and Health, School of Public Health, Qingdao University, Qingdao 266071, China; tailing1029@163.com (A.T.); lirongxu@qdu.edu.cn (L.X.); 2 National Center of Technology Innovation for Dairy, Hohhot 010110, China; szeto@yili.com (I.M.-Y.S.); wangxuemin1@yili.com (X.W.)

**Keywords:** docosahexaenoic acid, cognitive function, arachidonic acid, randomized controlled trials, meta-analysis

## Abstract

Objectives: Previous studies have assessed the effect of docosahexaenoic acid (DHA, 22:6*n*-3) and arachidonic acid (ARA, 20:4*n*-6)-supplemented infant formula on brain development and cognitive function in infants. However, the results have been inconsistent. The aim of this systematic review and meta-analysis was to assess the effect of DHA and ARA supplementation on cognitive function in infants from randomized controlled trials (RCTs). Methods: We systematically searched and identified relevant literature from the PubMed, Web of Science, and Embase databases up to July 2024. Standard methods were applied to assess publication bias, sensitivity analysis, and heterogeneity among the included studies. A total of nine RCTs were included in the study, which comprised 1039 subjects. Results: Meta-analysis showed significantly positive effects of DHA and ARA supplementation on cognitive development in infants (Standardized Mean Difference (SMD): 0.21; 95% CI: 0.03, 0.38). No significant difference was found in Mental Development Index (MDI) score (Weighted Mean Difference (WMD): 0.20; 95% CI: −0.03, 0.43) and Psychomotor Development Index (PDI) score (WMD: 0.12; 95% CI: −0.11, 0.35) in Bayley Scales of Infant and Toddler Development compared with the control group. In subgroup analysis, when DHA/ARA was 0.5–1, PDI had a significant difference (WMD: 0.48; 95% CI: 0.03, 0.93) compared with the control group, with no significant difference between heterogeneity (*I*^2^ = 46.4%, *p* = 0.155). In comparison to the control group, significant differences were observed in MDI when DHA/ARA levels were between 0.5 and 1 (WMD: 0.55; 95% CI: 0.07, 1.02), with no significant difference between heterogeneity (*I*^2^ = 51.6%, *p* = 0.127). Conclusion: When the DHA /ARA was 0.5–1 can significantly improve the cognitive function in infants.

## 1. Introduction

Long-chain polyunsaturated fatty acids (LCPUFAs) are a group of fatty acids with more than 2 double bonds and more than 19 carbon numbers [1], including omega-3 and omega-6 [2]. Their main functions include promoting neurodevelopment [3], regulating inflammation and promoting cardiovascular health [4]. Docosahexaenoic acid (C22:6*n*-3, DHA) and arachidonic acid (C20:4*n*-6, ARA) are synthesised from the essential fatty acids α-linolenic acid (ALA) and linoleic acid (LA), respectively, by liver-directed enzymatic pathways: ALA is progressively converted to EPA by FADS1/FADS2 desaturases and the elongases ELOVL5/ELOVL2, and then undergoes oxidative β-oxidation to produce DHA. LA produces ARA by the same enzyme system. Δ-6 Desaturase is the rate-limiting step, the efficiency of synthesis is very low (the conversion of ALA to DHA below 5% [5], the conversion of LA to ARA below 3% [6]), and it is regulated by age and diet, so it must be ingested directly (e.g., fish and breast milk). The brain and retina are dependent on exogenous DHA, and the placenta delivers it to the foetus via transporters. Both DHA and ARA were classified as long-chain polyunsaturated fatty acids, or PUFAs, which were essential in the development of infants [7]. DHA was classified as an omega-3 fatty acid, which was imperative for the optimal development of the brain, eyes, and nervous system in children [3]. In contrast, ARA is an omega-6 fatty acid that plays a crucial role in supporting normal brain development [8], as well as facilitating the repair and growth of muscle tissue [9], quickly accumulating in the central nervous system in the late period of pregnancy and the first year of life [10]. They are required for energy consumption and development, organogenesis and function, and cellular metabolism [7,11,12]. DHA (22:6*n*-3) is a product of the elongation/desaturation process from EFAs [13], α-linolenic acid (ALA). ARA (20:4*n*-6) is synthesized from its EFA precursor linoleic acid (LA) [14]. DHA influences the structure and functionality of cell membranes [15,16,17], and it holds lipid mediators with particular significance in the brain and retina, where it accumulates rapidly during the early stages of life [18,19]. Additionally, DHA is the precursor of specialized pro-resolving lipid mediators, such as resolvin, protectin, and maresin families, which are vital in preventing or treating prevalent chronic diseases that can lead to considerable morbidity and mortality [16,17]. The *n*-6 LCPUFAs, especially ARA, have extensively been found in human cells and tissues. Moreover, in the central nervous system, ARA is crucial for both structural and functional integrity, and is also a metabolic necessity for every cell, functioning as a precursor for eicosanoids that influence various biological processes, particularly those involved in cerebral, cardiovascular, and immune functions [20,21].

DHA supplementation has been shown to have potential health benefits in humans in a number of clinical trials [22,23,24]. During the developmental process, the conversion of linoleic acid to ARA by a desaturation step has been found to be inadequate in meeting nutritional requirements, particularly in carriers of the recently identified genetic variant of fatty acid desaturase [25]. This variant has been observed to weaken the biosynthetic production of ARA. Additionally, the study revealed that the levels of circulating DHA and ARA in breast-fed infants are only matched when both fatty acids were incorporated into the formula [26]. However, there have been limited studies on the effect of DHA and ARA supplementation on cognitive function in infants. A longitudinal, double-blind, controlled trial reported better cognitive development at age 24 months in infants that were fed a formula with DHA/ARA = 1/2 compared to those fed a formula with DHA/ARA = 1 [27]. Meanwhile, very low birth weight infants who received DHA and ARA had a positive effect on recognition memory and problem-solving scores than infants in the control group [28]. However, Devlin et al. [29] found that there was not a notable difference in cognition function among groups supplemented with different ratios of ARA to DHA. The previous published literature has inconsistent results, which may be due to different research designs, population characteristics, intervention duration and dosage and types. In addition, most of the studies were supplemented with DHA or ARA alone, and there are few articles on the combined supplementation effect of DHA and ARA on cognitive function, and it is not clear how much supplemental dose is better for cognitive development.

The addition of LCPUFAs in infant formula has sparked considerable debate. Research over the last decade has thoroughly examined the specific contributions of DHA and ARA to the development of healthy infants [19,30]. This subject has gained significant attention, especially given that 33% of newborns in numerous countries have been fed formula at birth, with this figure rising dramatically to 69% by the age of six months [31]. A key question emerges regarding the effectiveness of adding DHA and ARA to the formula in achieving the intended outcomes of enhanced infant development. It is crucial to understand the necessity of these LCPUFAs for optimizing the growth and development of neurological, cognitive, and visual functions in rapidly developing newborns. Supplementation of infant formulae with DHA alone, without ARA, may result in ARA deficiency due to limited endogenous synthesis. Infant Δ-6 desaturase activity is low (only 10–30% of adult activity in preterm infants) and DHA shares the enzyme with the ARA precursor (LA), causing competitive inhibition and further reducing the efficiency of ARA synthesis [32,33]. Clinical studies have shown that formulations lacking ARA can slow weight gain and reduce plasma ARA levels [34], while high DHA intakes can exacerbate ARA deficiency through substrate competition and enzyme inhibition [35].

It is important to note that a meta-analysis of the data from the included literature was conducted, including the ratio of DHA and ARA used in the infant intervention and their associations with cognitive performance from infancy. Breast milk, the gold standard for infant feeding, contains both DHA and ARA. Most studies performed to date have demonstrated that important physiological and developmental endpoints are sensitive to the ratio of dietary DHA /ARA. The aim of this meta-analysis was to review the evidence from published randomized controlled trials (RCTs) to evaluate the effects of DHA and ARA supplementation on cognitive development among infants. Subgroup analysis was conducted to assess the effects of intervention duration, region, ratio, and sample size on cognitive development outcomes. Ultimately, through an analysis of available RCT evidence, this study provided valuable insights for future nutritional supplementation strategies. This study determined the optimal ratio of DHA and ARA supplementation by subgroup analysis and provided a theoretical basis for the appropriate dosage of DHA and ARA supplementation in infant formula in the future.

## 2. Methods

### 2.1. Literature Search

A comprehensive literature review was performed through July 2024, utilizing the PubMed, Web of Science and Embase databases. “Fatty acid and cognition”, “Fish oil and cognition”, “Omega-6 and Omega-3 and cognition”, “Docosahexaenoic acid (DHA) and Arachidonic Acid and cognition” were keywords. The full details of the search strategy are displayed in Table S1. Additionally, the reference lists from original research articles, reviews, and meta-analyses were examined using Google Scholar and Baidu Scholar. Prior to the study selection, the protocol of this study was registered through the International Prospective Register of Systematic Reviews in PROSPERO (no. CRD42024578904).

### 2.2. Inclusion and Exclusion Criteria

The search strategy was employed to obtain the article titles, which were then subjected to a thorough examination, encompassing the titles, abstracts, and full texts. This process was undertaken to ascertain whether the articles should be included or excluded. The PICO criteria for the current meta-analysis were as follows: intervention (DHA and ARA; Long-chain unsaturated fatty acids containing DHA and ARA into the intervention, DHA and ARA as the primary compositional distinctions); comparison (control or placebo group); outcomes (cognition development); furthermore, observational studies, case reports, etc., were excluded. In addition, articles containing *n*-6 and *n*-3 interventions without specific levels of DHA and ARA were excluded. The comprehensive overview of the inclusion and exclusion criteria is shown in Table 1.

### 2.3. Cognitive Performance Measures

The articles included in this meta-analysis utilized Bayley Scales of Infant and Toddler Development (BSID) and Brunet–Lezine developmental quotient (DQ) test as assessment tools for cognitive function evaluation and developmental score. In this paper, forest plots were generated based on the average and standard deviation of the Mental Development Index (MDI) and Psychomotor Development Index (PDI) calculated using the BSID and DQ for data analysis and processing, aiming to assess the overall impact of different ratios of DHA and ARA on cognitive function. Cognitive outcomes were measured using Standardized Mean Difference (SMD), PDI and MDI were measured using Weighted Mean Difference (WMD) Given the heterogeneity in cognitive assessment scales used across the studies, this article adopted the SMD approach to ameliorate the effects of scale variability on the cognitive measures for which baseline data were accessible. For those studies that did not provide such foundational data, the study resorted to the WMD method, supplemented by the Baley scale for cognitive assessments.

### 2.4. Data Extraction and Quality Assessment

Data extraction was carried out independently by two different investigators, and any disagreements were resolved through discussions leading to a consensus. The fundamental details of the selected articles were collected, which included the first author’s surname, year of publication, country of origin, gender, sample size, age range, intervention duration, and types of interventions employed. For the studies that were included, the means and standard deviations (SDs) of PDI, MDI, and DQ at the endpoint for both the control and intervention groups were retrieved separately. The final outcome data were gathered if the trial reported results multiple times across different stages. In cases where the SD was not supplied in the trial, we computed it using the interquartile range or the standard error of the mean (SEM), based on the formula provided in the Cochrane Handbook [36]. Similarly, the risk of bias in randomized trials was evaluated using the Cochrane Handbook.

### 2.5. Statistical Analysis

Review Manager 5.3 (Cochrane Collaboration, Oxford, UK) and Stata 17 (Stata Corp., College Station, TX, USA) were utilized for statistical analysis. All variables involved in the study were continuous; therefore, the WMD and SMD, expressed as a 95% confidence interval (CI), were employed to calculate the overall effect size of the intervention. The mean and SD of the PDI and MDI were estimated in summary using the random effects model developed by DerSimonian and Laird [37]. Heterogeneity across selected trials was assessed using the *I*^2^ test and divided into high (*I*^2^ > 50%), medium (25%< *I*^2^ ≤50%), and low degree (*I*^2^ ≤ 25%) heterogeneity. *p* < 0.05 was considered statistically significant. We focused on trial-related information: region, mean duration, ARA or DHA supplementation dose, study duration, and ethnic differences. To assess whether the overall effect was stable, we performed sensitivity analysis by deleting one trial at a time and recalculating the effect size. The funnel plot and Egger’s test were used to estimate the possibility of publication bias (significant at *p* < 0.05).

## 3. Results

### 3.1. Study Selection

**Figure 1:**
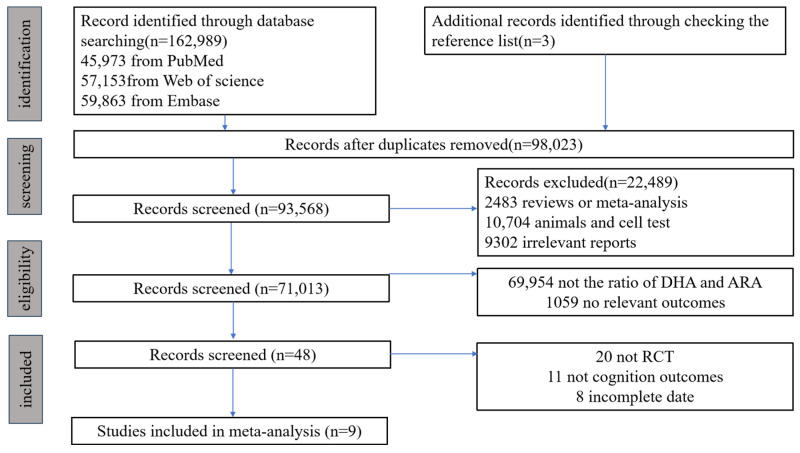
Literature search and review flowchart for selection of studies.

Figure 1 illustrates a flowchart depicting the process of searching and selecting studies, after searching the PubMed, Web of Science, and Embase databases. In addition to conducting manual searches, a total of 26,953 relevant RCTs were identified from July 2024. After eliminating duplicate articles, 12,933 publications remained. Further screening of titles and abstracts led to the inclusion of 3444 publications, filtering out irrelevant reports, article types, and research subjects. Following this, 48 potentially relevant articles were selected after discarding reports that were unrelated to DHA/ARA or lacked pertinent outcomes. Subsequently, 39 articles were excluded due to not being RCTs or having incomplete data. Ultimately, 9 publications were determined to be suitable for the current meta-analysis (Figure 1).

### 3.2. Study Characteristics

Table 2 shows a brief introduction to the nine articles included in the study [28,29,30,38,39,40,41,42,43]. The data from this literature were collected and analyzed to draw the forest plots for the meta-analysis. Nine reports published in 1995 or later were identified. Of these, two original studies had subsequent reports at ages 4 and 24 months. The authors extracted samples of infants at 4 months and 24 months of age to measure the DQ value of infants. In the study of Westerberg [28], the control group received the same mixture of soy oil and medium-chain triglyceride oil as the study group, but without DHA and ARA. In Devlin’s study [29], the control group received corn oil supplements without DHA and ARA. In the other seven articles, all control groups were fed formula without DHA and ARA. In six articles, the proportion of DHA in the total fatty acids was 0.3% to 0.4%. In the other three articles [29,40,43], the proportion of DHA in total fatty acids was 0.1% to 0.2%. In nine articles, the proportion of ARA in the total fatty acids was between 0.12% and 0.72%. Because of the competitive inhibition of DHA and ARA, different proportions of DHA and ARA may have different effects on cognitive function in infants. The characteristics of the included articles are shown in Table 2. There were differences in the intervention duration, the dose of the intervention, and the regions of the intervention among the nine articles. Therefore, it was essential to conduct the meta-analysis to evaluate the effects of DHA and ARA supplementation on cognitive development among infants in this study.

In the cohort of newborns analyzed in the research, eight studies focused on healthy-term infants, while one study [27] examined very low birth-weight babies. The majority of these infants were single births and suitable for their gestational age. Infants suspected of having a milk protein intolerance, or those with a familial history of such intolerance, were excluded from nine trials. To be eligible for participation in the trial, most studies required participants to have had an uncomplicated pregnancy and to be free of congenital diseases or prior experiences in a neonatal intensive care unit. Among the studies reviewed, four studies were conducted in North America [29,30,40,43], two studies in Europe [28,38,39,41], and the remaining one study was in Oceania [42]. All the studies provided reasons for excluding certain participants. Only two trials reported household smoking, which was known to influence fetal as well as postnatal growth and health [28,41].

### 3.3. Risk of Bias Assessment

**Figure 2:**
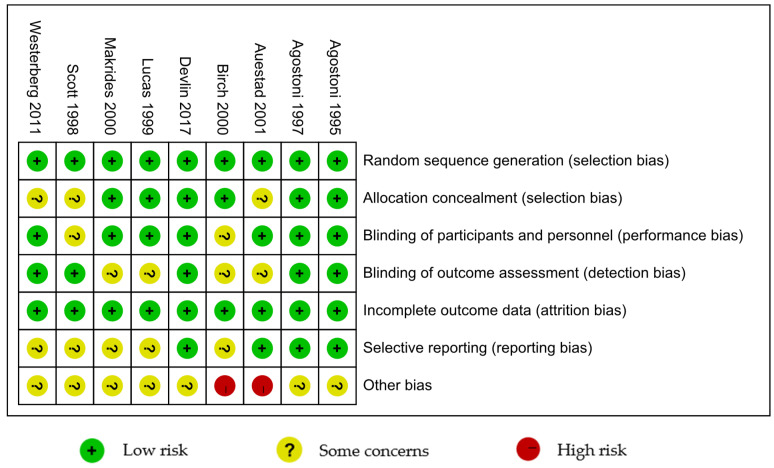
Risk of bias summary reviews authors’ judgements about each risk of bias item for each included study [28,29,30,38,39,40,41,42,43].

**Figure 3:**
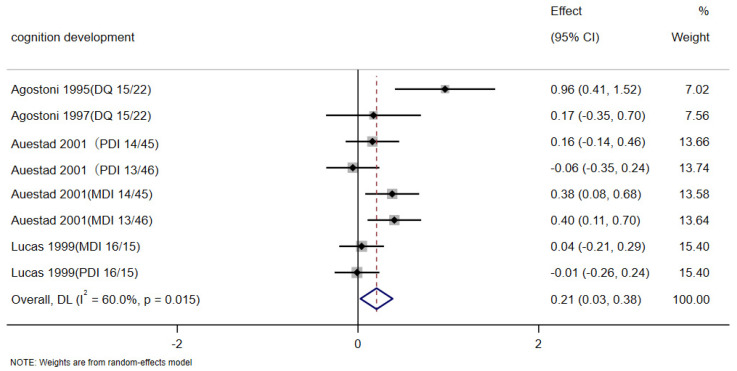
Forest plots of the effects of DHA and ARA supplementation on the change of cognitive development. Abbreviations: DQ, Developmental quotient; PDI, Psychomotor Development Index; MDI, Mental Development Index. The content in parentheses following the reference refers to DHA/ARA [38,39,41,43].

Cochrane’s bias risk assessment concluded that seven out of the selected articles had low and moderate bias risks, respectively, as shown in Figure 2 and Figure 3. Two studies [28,43] were found to have high bias risks because of their inclusion of multiple sources of bias.

### 3.4. The Effect of DHA and ARA Supplementation on Cognitive Development in Infants

**Figure 4:**
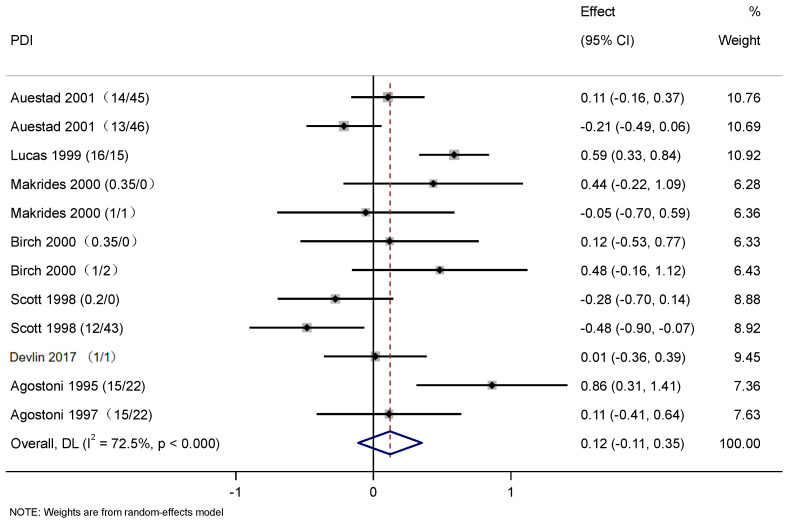
Forest plots of the effects of DHA and ARA supplementation on the change of Psychomotor Development Index score among infants. Abbreviations: PDI, Psychomotor Development Index;. The content in parentheses following the reference refers to DHA/ARA [29,30,38,39,40,41,42,43].

**Figure 5:**
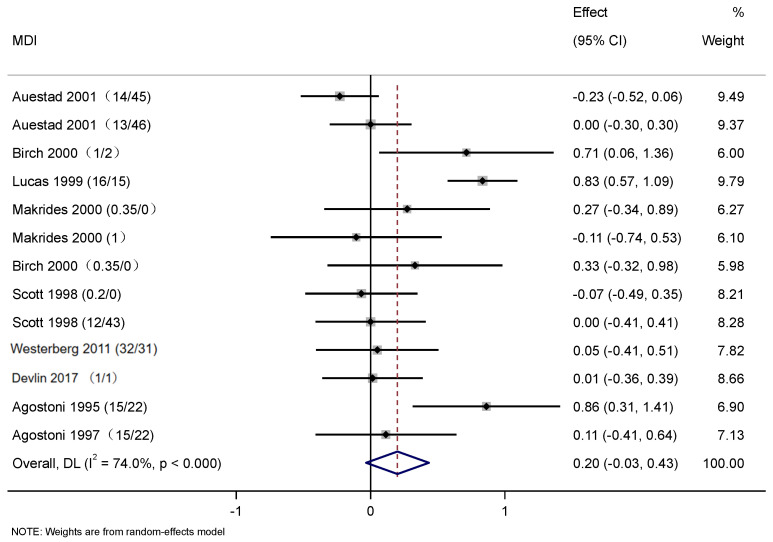
Forest plots of the effects of DHA and ARA supplementation on the change of Mental Development Index score among infants. Abbreviations: MDI, Mental Development Index. The content in parentheses following the reference refers to DHA/ARA [28,29,30,38,39,40,41,42,43].

A total of nine studies were used to evaluate the effect of DHA and ARA supplementation on cognitive development. For the four articles included in Figure 3, baseline data were measured prior to the commencement of the intervention. Following the intervention, SMD values were calculated before and after the intervention for meta-analysis, thereby ensuring the results were more accurate and realistic. Cognitive development presented a significant difference between the control and intervention groups (SMD: 0.21, 95% CI: 0.03, 0.38; *p* < 0.05). The heterogeneity was high (*I*^2^ = 60.0%; *p* < 0.05) (Figure 3). The results showed significantly positive effects of DHA and ARA supplementation on cognitive development in infants. Figure 4 used PDI data to plot the forest, while Figure 5 used MDI to plot the forest. In Figure 4, eight articles were used to evaluate the effects of DHA and ARA supplementation on the change of PDI score among infants (8 trials, 634 cases, and 595 controls). In the control and intervention groups, there was no significant difference in PDI concentrations (WMD: 0.12, 95% CI: −0.11, 0.35), and heterogeneity was high (*I*^2^ = 72.5%, *p* < 0.001) (Figure 4). In Figure 5, there are nine articles to evaluate the effects of DHA and ARA supplementation on the change of MDI score among infants (9 trials, 599 cases, and 635 controls). In the control and intervention groups, there was no significant difference in MDI concentrations (WMD: 0.20, 95% CI: −0.03, 0.43), and heterogeneity was high (*I*^2^ = 74.0%, *p* < 0.001) (Figure 5). There was no significant difference in PDI and MDI, which can be attributed to mean age, regions, intervention duration, type of DHA and ARA; therefore, subgroup analysis was performed.

### 3.5. Subgroup Analysis

**Figure 6:**
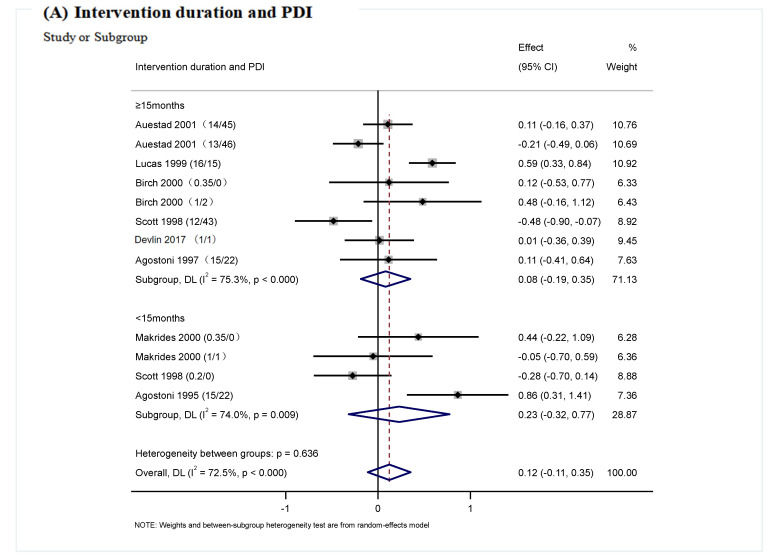

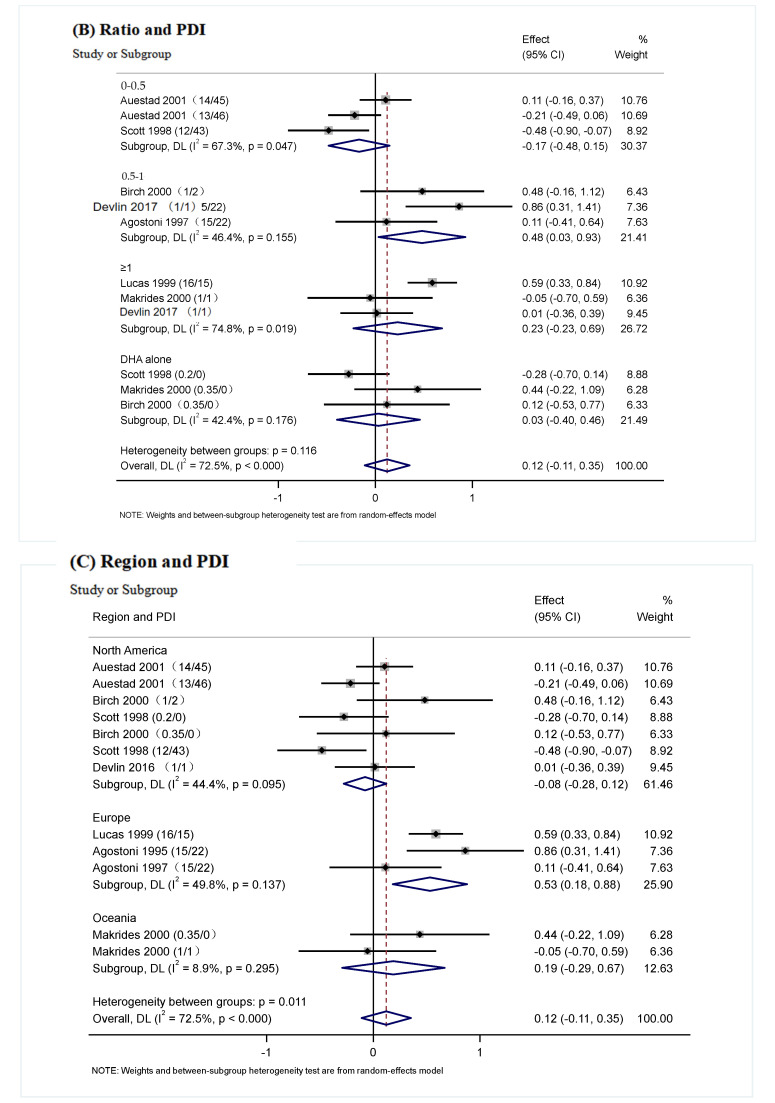

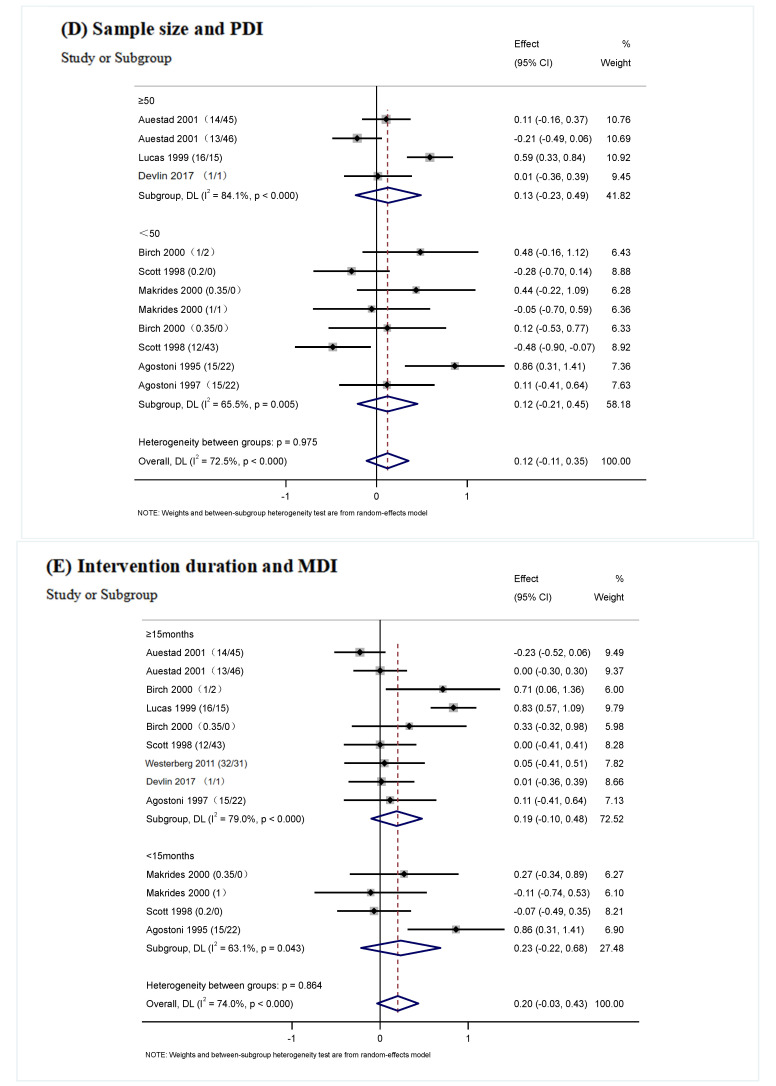

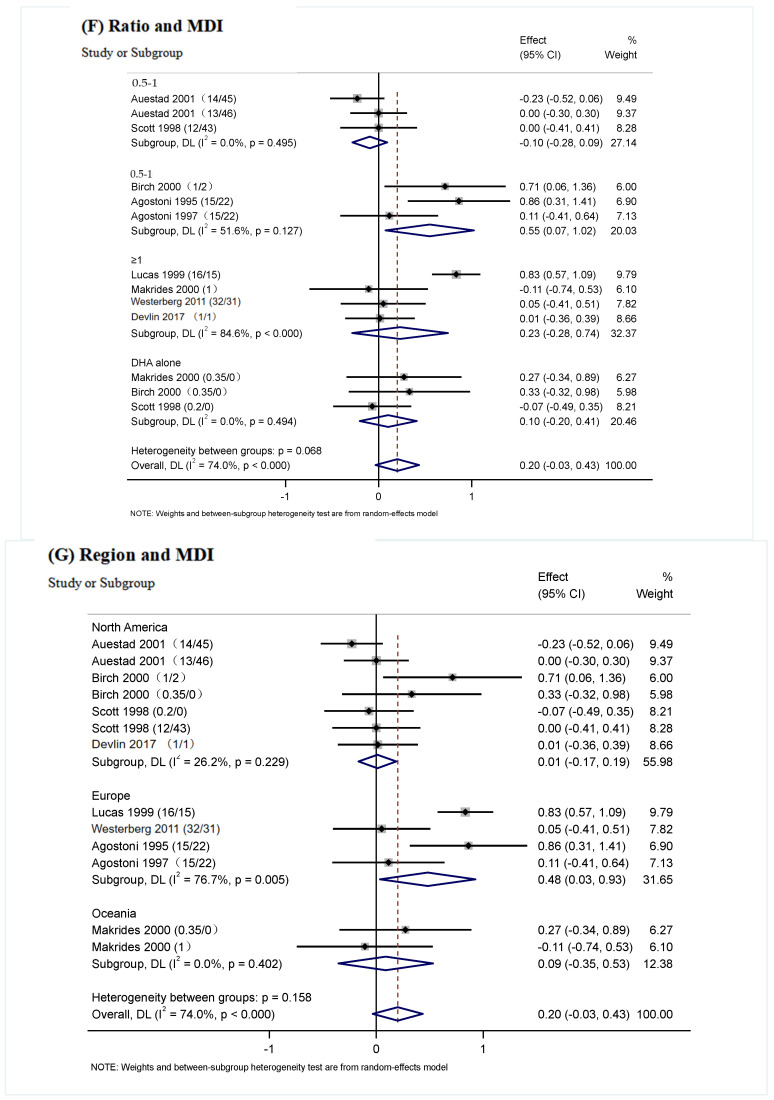

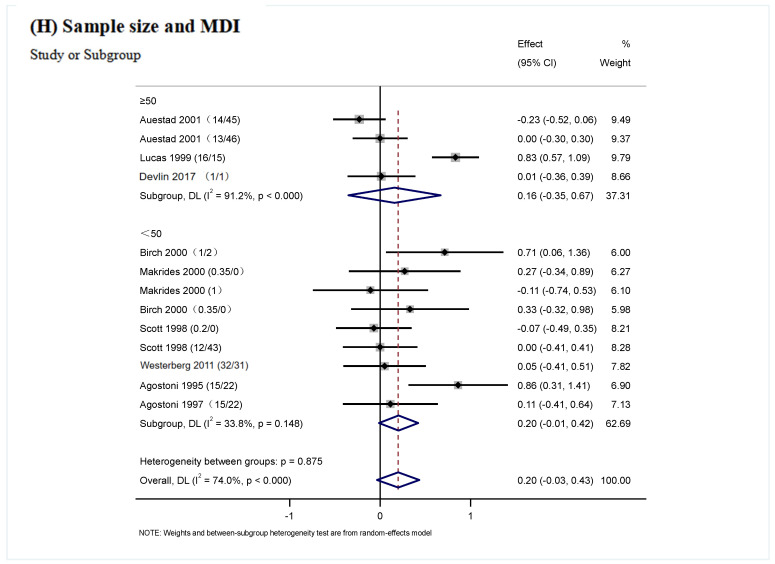
Subgroup analysis of the effects of DHA and ARA supplementation on cognition development. ((**A**): the effect of Intervention duration on PDI; (**B**): the effect of the ratio of Intervention on PDI; (**C**): the effect of region on PDI; (**D**): the effect of sample size on PDI; (**E**): the effect of Intervention duration on MDI; (**F**): the effect of the ratio of Intervention on MDI; (**G**): the effect of region on MDI; (**H**): the effect of sample size on MDI). Abbreviations: PDI, Psychomotor Development Index; MDI, Mental Development Index. The content in parentheses following the reference refers to DHA/ARA [28,29,30,38,39,40,41,42,43].

In this study, subgroup analysis was performed, with the largest ratio of 16/15 in the study of DHA and ARA supplementation, according to different intervention times (Figure 6A,E), intervention ratio (Figure 6B,F), study region (Figure 6C,G), and sample size (Figure 6D,H). Twelve trials reported no significant effect of DHA and ARA on PDI (WMD: 0.12; 95%CI: −0.11, 0.35, *I*^2^ = 72.5%, *p* = 0.116); however, the effect of DHA/ARA at 0.5–1 was found to be significant (WMD: 0.48; 95% CI: 0.03, 0.93), and the heterogeneity was not statistically significant (*I*^2^ = 46.4%, *p* = 0.155) (Figure 6B). Thirteen trials reported no significant effect of DHA and ARA supplementation on MDI (WMD: 0.20; 95%CI: −0.03, 0.41, *I*^2^ = 74.0%, *p* = 0.068). Conversely, the effect of DHA and ARA at a ratio from 0.5 to 1 was found to be statistically significant (WMD: 0.55; 95% CI: 0.07, 1.02) with no statistically significant heterogeneity (*I*^2^ = 51.6%, *p* = 0.127) (Figure 6F). In terms of PDI, when the analysis was grouped by region, DHA and ARA intervention had a significantly positive effect on the cognitive development of infants from Europe (WMD: 0.53; 95% CI: 0.18, 0.88, *I*^2^ = 49.8%, *p* = 0.137) (including Italy, the Netherlands, Norway and the United Kingdom) compared with infants from other regions (Figure 6C). In terms of MDI, when the analysis was grouped by region, DHA and ARA intervention had a significantly positive effect on the cognitive development of infants from Europe (WMD: 0.48; 95% CI: 0.03, 0.93, *I*^2^ = 76.7%, *p* = 0.005) compared with infants from other regions (Figure 6G). In addition, the effect of different intervention times and sample size on cognitive function had no significant differences in subgroup analysis, the detailed information was shown in Table 3 and Table 4.

### 3.6. Sensitivity Analysis

The impact of each individual trial was explored by removing each study one at a time from the analysis (Figure S1), and we found that no single trial significantly influenced the overall effect size of cognitive development.

### 3.7. Publication Bias

Based on the examination of funnel plots and the results of Egger’s regression test, it was determined that there was no significant publication bias present in the research focusing on the effects of DHA and ARA supplementation on the Mental Development Index and Psychomotor Development Index scores (Egger’s *p* values = 0.5906 and 0.5236, respectively). Although the funnel plots related to cognitive development exhibited a slight asymmetry, this indicated a potential minimal risk of bias (Figure S2).

## 4. Discussion

This meta-analysis of RCTs was the first to systematically evaluate the effects of DHA and ARA supplementation on the cognitive development of infants. This study showed that DHA and ARA intake had a significantly positive effect on cognitive development levels yet did not affect PDI and MDI levels. The DHA /ARA at 0.5–1 supplementation improved overall clinical features. The previous reviews assessing the safety and advantages of adding LCPUFAs to the formula for full-term infants were examined, with an emphasis on impact on visual function, neurodevelopment, and physical growth [44,45]. One notable concern regarding the inclusion of LCPUFA, particularly DHA alongside ARA, was its potential to influence infant growth. Research involving infants with very low body weight indicated that LCPUFA derived from fish oil was associated with a reduction in ARA levels in the membranes of red blood cells (RBCs), as well as reduced growth rates [46]. This suggests that fatty acid supplementation should not be excessive, as surpassing a certain threshold could pose risks to human health [47]. Consequently, the necessity of adding ARA to formulas that already contain DHA has been questioned. For this reason, three [30,40,42] of the nine studies implemented various treatment groups: one group received a standard formula, another group was given a formula enriched solely with DHA, and a third group received a formula that included both DHA and ARA.

This study focused on cognitive development. The elementary forms of cognitive functions like attention and memory start to develop during a relatively young age, whereas more advanced, goal-directed behaviours, including strategic planning, reasoning, and other executive functions, appear at later stages of development [48,49]. Commonly used assessment instruments, such as the BSID, assess behavioural responses (encompassing both mental and motor development) to evaluate whether these responses lag, align with, or exceed a typical developmental timeline.

The cognitive scales selected in the SMD in Figure 3 included BSID and the Brunet–Lezine developmental quotient test. This Brunet–Lezine developmental quotient test was developed in France [50]. According to this scale, scores above 90 indicated normal functioning, 80–90 score indicated below average functioning, 70–79 score indicated borderline functioning, and below 70 score constitute mental retardation. Brunet–Lezine quotients correspond roughly (based on clinical experience) to more standardized intelligence test scores.

In this paper, these particular tests were more apt to detect the impacts of nutrients (or environmental factors) on the developing infant compared to general assessment tools, as certain cognitive abilities may be distinctly influenced by LCPUFAs [51]. The BSID and the Brunet–Lezine developmental quotient test were used to measure cognitive outcomes in infants. Among them, seven articles [28,29,30,40,41,42,43] used the BSID, and two articles [38,39] used the Brunet–Lezine developmental quotient test. The study chose different calculation methods according to different data, and the measurement of outcomes was more scientific and reasonable.

Despite the fact that the significance of fatty acids for human health and well-being was acknowledged nearly 90 years ago [51,52], it was in the past 30 years that substantial interest has emerged in comprehending the roles of LCPUFAs in the growth and development of infants [16,17,53,54]. The foundational study by Martinez, which illustrated the swift accumulation of DHA and ARA in the infant’s brain throughout the initial 1000 days of life [18], motivated numerous researchers to engage in this crucial field of study [55,56]. The incorporation of DHA and ARA in infant formulas has been the subject of scientific discussions. In this regard, in the USA and other countries, the addition of DHA and ARA in infant formulas is mandatory, while in the European Union, only the addition of DHA is mandatory. It reflects the controversy in the scientific community about the necessity and safety of ARA. DHA is widely recognised for its strong evidence of neurodevelopment [57], while ARA supports membrane structure and immune balance [58], but its pro-inflammatory potential and the difference in ratio to human milk have raised concerns [59]. The US FDA supports the addition of ARA based on short-term cognitive improvement studies, while the EU EFSA believes that infants can synthesise ARA endogenously and that long-term safety data are lacking [60].

This systematic review and meta-analysis examined the effect of DHA and ARA supplementation on cognitive development. The study drew forest plots to evaluate the effects of different proportions of DHA and ARA on the cognitive outcomes of infants using different cognitive measures (SMD and WMD). The studies using SMD measures of cognitive outcomes showed that the addition of DHA and ARA in the intervention has a positive effect on the cognitive outcomes of infants. The includes studies using WMD measures of cognitive outcomes, which found no significant difference between infants receiving DHA or ARA supplementation and those receiving no supplementation. Due to the different references included, the limited data available, and the different scales used for cognitive assessment, the results using two different cognitive measures from Figure 4, Figure 5 and Figure 6 emerged.

Recently, most of the literature on brain and cognitive function has focused on DHA; however, ARA has also been implicated in central nervous system function [7,48,61], and *n*-6 LCPUFAs are also important in the developing primate rain [9]. Therefore, it is vital to study the effect of DHA and ARA on cognitive function. A previous article found that the DHA/ARA of 1.5:1 reduced ARA in the erythrocyte membrane compared to a 1:2 or 1:1 ratio [53]. Because of the reductions in ARA and other *n*-6 LCPUFAs found in the brains of developing baboons fed 1.5:1 to 1:2 of DHA to ARA, they suggest that similar reductions may occur in the brains of infants. Colombo et al. [62] found that the balance of DHA and ARA in formula may be a factor contributing to the benefit of LCPUFA supplementation during infancy and early childhood. Neurodevelopmental outcomes support a DHA/ARA of 1:1 or 1:2, which is consistent with the literature included in our study.

This study used two methods (SMD and WMD) to make three forest plots to analyze the effects of different proportions of DHA and ARA on cognition in infants, due to the inconsistency of cognitive function measurement methods, including the use of Brunet–Lezine developmental quotient and Bayley Scales of Infant and Toddler Development. Changes in values before and after the intervention were analyzed by us, and the forest plot results showed that the intervention group had a significant improvement in cognitive function scores compared with the control group (Figure 3). It was concluded that the addition of the intervention does improve the cognitive function of infants. Later, it was found several articles that used BSID as the scoring standard of cognitive function for analysis, as shown in Figure 5 and Figure 6, and no significant differences were found. The possible reasons included racial differences, differences in the dosage of interventions, and differences in the duration of interventions. More researchers were needed to participate in the study, standardize the dose and time of intervention, and improve the research protocol for the development of cognitive function.

From the literature included, these infants were randomly assigned to receive formula containing DHA and ARA with intervention ratios between 0–16/15. The study conducted subgroup analysis of different intervention times, intervention doses, different regions, and sample sizes, and found that the intervention dose and the region had significant effects on cognitive function. The PDI results showed that the effect on cognitive function was significant when the intervention DHA/ARA was 0.5–1. The results of the MDI found a significant difference between the intervention group and the control group when the DHA/ARA was 0.5–1. Previous experiments have shown that when the DHA/ARA was too high, it produces competitive inhibition in the body, which would affect the improvement of cognitive function [62]. Our research results revealed that when DHA/ARA was 0.5–1, a significant difference was found for MDI and PDI: it had a better improvement effect on cognitive function. Grouped by region, it was found that the DHA/ARA supplementation had significant differences in the European population compared with other countries in North America and Oceania, and our results suggest that the European population is more likely to benefit from DHA/ARA at 0.5–1 supplementation in the future.

This study demonstrated that an optimal DHA/ARA ratio of 0.5–1 exhibits significantly positive effects on cognitive development in infants.

### Strengths and Weakness

RCTs are considered the highest level of evidence to establish causal associations in clinical research. In contrast, observational studies are generally considered to provide lower-quality evidence due to the increased risk of bias associated with the absence of randomization and the investigators’ inability to control for known or unknown confounding factors. All included studies were RCTs, which lends strength to the results.

Despite the strict inclusion criteria and rigorous methodology, some limitations must be recognized. The limitations include the fact that the concentration of DHA and ARA in infant formulas was inconsistent (from 0% to 0.41% of DHA and from 0% to 0.72% of ARA). There was a difference in formula feeding time for infants (4–24 months). When making a cognitive assessment of infants, the age does not match the brain’s development (18 months may not be an appropriate assessment time, since a baby’s brain changes rapidly) [63]. Assessment of research utilizing the BSID and Brunet–Lezine developmental quotient test in this meta-analysis may lead to heterogeneity among these studies, which limits the effectiveness of the meta-analysis. Furthermore, the literature included in this meta-analysis was published prior to the current study, and there are few recent studies, which represents another limitation of this paper.

## 5. Conclusions

This meta-analysis provided substantial evidence that DHA and ARA supplementation can improve the level of cognitive function in infant subjects to some extent. Meta-analysis showed significantly positive effects of DHA and ARA supplementation on cognitive development in infants. The results of our subgroup analysis showed a positive effect on PDI and MDI when the intervention DHA/ARA was 0.5–1. It is recommended to keep the DHA/ARA intervention between 0.5–1 for infants. The study suggests that the combination of DHA and ARA is superior to DHA or ARA supplementation alone and that strict attention should be paid to the DHA/ARA. However, considering the limitations of our meta-analysis mentioned above and the insufficient number of studies in the existing literature, the findings must be interpreted with care. In the future, additional RCTs should be carried out to offer clearer evidence regarding the relative effectiveness of various DHA/ARA formulations. Meta-analysis supports maintaining a DHA/ARA of 0.5–1 in infant formulas to enhance cognitive outcomes. This study aims to provide a comprehensive overview of the nutritional value of DHA and ARA supplementation in infants. In addition, it seeks to offer a theoretical foundation for the future enhancement of formula powder).

## Figures and Tables

**Table 1:** Summary of inclusion and exclusion criteria.

	Inclusion Criteria	Exclusion Criteria
Participants	Infants	Mothers or infants with defined diseases or disorders
Interventions	DHA and ARA Long-chain unsaturated fatty acids containing DHA and ARA into the intervention, DHA and ARA as the primary compositional distinctions.	In combination with drugs, nutrients, or other interventions The principal distinction between the interventions was not the inclusion of DHA and ARA
Comparators	Placebo or no intervention	
Outcomes of interest	Cognition development	Uncorrelated results
Study design	Randomized controlled study	Non-randomized/uncontrolled/observational studies (cross-sectional, case-control, and cohort) Animal models, in vitro, in vivo, ex-vivo trials, or quasi-experimental studies
Publications	English articles and original research	Non-original research (commentaries, editorials, or reviews), duplicated studies, unpublished studies, abstracts Published as conference proceedings Languages other than English

**Table 2:** Basic information of the included data.

Author (Year)	Study Groups	DHA Percentage of Total Fatty Acid	ARA Percentage of Total Fatty Acid	DHA/ARA	Evaluations, Outcome		Region	Intervention Duration
Westerberg 2011 [28]	G1 N = 44	0	0	0	BSID (including MDI)	-	Europe	20 months
	G2 N = 48	0.32%	0.31%	32/31				
Devlin 2017 [29]	G1 N = 58	0	0	0	BSID (including MDI)	BSID (including PDI)	North America	24 months
	G2 N = 52	0.12%	0.12%	1/1				
Birch 2000 [30]	G1 N = 20	0	0	0	BSID (including MDI)	BSID (including PDI)	North America	18 months
	G2 N = 17	0.35%	0	0.35/0				
	G3 N = 19	0.36%	0.72%	1/2				
Agostoni 1995 [38]	G1 N = 27	0.30%	0.44%	15/22	Brunet–Lezine (including MDI)	Brunet–Lezine (including PDI)	Europe	4 months
	G2 N = 29	0	0	0				
Agostoni 1997 [39]	G1 N = 26	0.30%	0.44%	15/22	Brunet–Lezine (including MDI)	Brunet–Lezine (including PDI)	Europe	24 months
	G2 N = 29	0	0	0				
Scott 1998 [40]	G1 N = 42	0	0	0	BSID (including MDI)	BSID (including PDI)	North America	12 months
	G2 N = 33	0.20%	0	0.2/0				
	G3 N = 38	0.12%	0.43%	12/43				
Lucas 1999 [41]	G1 N = 125	0	0	0/0	BSID (including MDI)	BSID (including PDI)	Europe	18 months
	G2 N = 125	0.32%	0.30%	16/15				
Makrides 2000 [42]	G1 N = 21	0	0	0	BSID (including MDI)	BSID (including PDI)	Oceania	18 months
	G2 N = 23	0.35%	0	0.35/0				
	G3 N = 24	0.34%	0.34%	1/1				
Auestad 2001 [43]	G1 N = 77	0	0	0	BSID (including MDI)	BSID (including PDI)	North America	6, 12 months
	G2 N = 80	0.14%	0.45%	14/45				
	G3 N = 82	0.13%	0.46%	13/46				

BSID, Bayley Scales of Infant and Toddler Development; PDI, Psychomotor Development Index; MDI, Mental Development Index.

**Table 3:** Subgroup analysis of the effects of DHA and ARA supplementation on PDI.

Variables	Subgroups	Number of Effect Sizes	WMD (95% CI)	*p* ^a^	Test for Subgroup Difference *p*	*I* ^2^	*p* ^b^
Intervention duration	>15 months	8	0.08 (−0.19, 0.35)	*p* < 0.001	*p* = 0.22	75.3%	*p* = 0.636
	≤15 months	4	0.23 (−0.32, 0.77)	*p* = 0.009	*p* = 0.46	74.0%	
The ratio of Intervention (DHA/ARA)	0–0.5	3	−0.17 (−0.48, 0.15)	*p* = 0.047	*p* = 0.16	67.3%	*p* = 0.116
	0.5–1	3	0.48 (0.03, 0.93)	*p* = 0.155	*p* = 0.04	46.4%	
	≥1	3	0.23 (−0.23, 0.69)	*p* = 0.019	*p* = 0.32	74.8%	
	DHA alone	3	0.03 (−0.40, 0.46)	*p* = 0.176	*p* = 0.90	42.4%	
Region	North America	7	−0.08 (−0.28, 0.12)	*p* = 0.095	*p* = 0.44	44.4%	*p* = 0.011
	Europe	3	0.53 (0.18, 0.88)	*p* = 0.137	*p* =0.003	49.8%	
	Oceania	2	0.19 (−0.29, 0.67)	*p* = 0.295	*p* = 0.44	8.9%	
Sample size	≥50 persons	4	0.13 (−0.23, 0.49)	*p* < 0.000	*p* = 0.48	84.1%	*p* = 0.975
	<50 persons	8	0.12 (−0.21, 0.45)	*p* = 0.005	*p* = 0.48	65.5%	

Abbreviations: *p* ^a^ for heterogeneity; *p* ^b^ for meta-regression analysis.

**Table 4:** Subgroup analysis of the effects of DHA and ARA supplementation on MDI.

Variables	Subgroups	Number of Effect Sizes	WMD (95% CI)	*p* ^a^	Test for Subgroup Difference *p*	*I* ^2^	*p* ^b^
Intervention duration	>15 months	9	0.19 (−0.10, 0.48)	*p* < 0.000	*p* = 0.15	79.0%	*p* = 0.864
	≤15 months	4	0.23 (−0.22, 0.68)	*p* = 0.043	*p* = 0.32	63.1%	
The ratio of Intervention (DHA/ARA)	0–0.5	3	−0.10 (−0.28, 0.09)	*p* = 0.495	*p* = 0.32	0.0%	*p* = 0.068
	0.5–1	3	0.55 (0.07, 1.02)	*p* = 0.127	*p* = 0.02	51.6%	
	≥1	4	0.23 (−0.28, 0.74)	*p* < 0.000	*p* = 0.38	84.6%	
	DHA alone	3	0.10 (−0.20, 0.41)	*p* = 0494	*p* = 0.52	0.0%	
Region	North America	7	0.01 (−0.17, 0.19)	*p* = 0.229	*p* = 0.46	26.2%	*p* = 0.158
	Europe	4	0.48 (0.03, 0.93)	*p* = 0.005	*p* = 0.04	76.7%	
	Oceania	2	0.09 (−0.35, 0.53)	*p* = 0.402	*p* = 0.70	0.0%	
Sample size	≥50 persons	4	0.16 (−0.35, 0.67)	*p* < 0.000	*p* = 0.54	91.2%	*p* = 0.875
	<50 persons	9	0.20 (−0.01, 0.42)	*p* = 0.148	*p* = 0.07	33.8%	

Abbreviations: *p* ^a^ for heterogeneity; *p* ^b^ for meta-regression analysis.

## Data Availability

The datasets used or analyzed during the current study are available from the corresponding author upon reasonable request.

## Supplementary Materials

The following supporting information can be downloaded at: https://www.mdpi.com/article/10.3390/nu17061091/s1. Table S1. Literature search strategy in PubMed. Table S2. Literature search strategy in web of science. Table S3. Literature search strategy in EMBASE. Figure S1. (A) Sensitivity analysis of cognitive function. (B) Sensitivity analysis of PDI. (C) Sensitivity analysis of MDI. Figure S2. (A) Funnel plot for assessing publication bias on cognition development. (B) Funnel plot for assessing publication bias on PDI. (C) Funnel plot for assessing publication bias on MDI.

## Abbreviations

DHA	Docosahexaenoic acid
ARA	Arachidonic acid
BSID	Bayley Scales of Infant and Toddler Development
MDI	Mental Development Index
PDI	Psychomotor Development Index
DQ	Developmental quotient
RCT	Randomized clinical trial
CI	Confidence interval
WMD	Weighted mean difference
SMD	Standardized mean difference
LCPUFAs	Long-chain polyunsaturated fatty acids
