# Supplementary material for: Effects of Different Proportions of DHA and ARA on Cognitive Development in Infants: A Meta-Analysis
Source: Nutrients. 2025 Mar 20;17(6):1091. doi: 10.3390/nu17061091 (PMC11946645; doi:10.3390/nu17061091)
Supplement: Supplementary file 1 [file nutrients-17-01091-s001.zip › nutrients-3506703-supplementary.pdf]

Effects of Different Proportions of DHA and ARA on Cognitive Development in Infants: A Meta-Analysis

Supplementary data including 3 Tables and 2 Figures

Table S1. Literature search strategy in PubMed.

1	"Cognition"[Mesh]
2	((((Cognitions) OR (Cognitive Function)) OR (Cognitive Functions)) OR (Function, Cognitive)) OR (Functions, Cognitive)
3	"Fatty Acids"[Mesh]
4	((((((((((((Fatty Acid) OR (Aliphatic Acids)) OR (Aliphatic Acid)) OR (Acid, Aliphatic)) OR (Fatty Acids, Esterified)) OR (Esterified Fatty Acids)) OR (Esterified Fatty Acid)) OR (Acid, Esterified Fatty)) OR (Fatty Acid, Esterified)) OR (Fatty Acids, Saturated)) OR (Saturated Fatty Acids)) OR (Saturated Fatty Acid)) OR (Acid, Saturated Fatty)) OR (Fatty Acid, Saturated)
5	(((((((((((((Fatty Acid) OR (Aliphatic Acids)) OR (Aliphatic Acid)) OR (Acid, Aliphatic)) OR (Fatty Acids, Esterified)) OR (Esterified Fatty Acids)) OR (Esterified Fatty Acid)) OR (Acid, Esterified Fatty)) OR (Fatty Acid, Esterified)) OR (Fatty Acids, Saturated)) OR (Saturated Fatty Acids)) OR (Saturated Fatty Acid)) OR (Acid, Saturated Fatty)) OR (Fatty Acid, Saturated)) AND (((((Cognitions) OR (Cognitive Function)) OR (Cognitive Functions)) OR (Function, Cognitive)) OR (Functions, Cognitive))
6	"Fish Oils"[Mesh]
7	((((((Oils, Fish) OR (Fish Oil)) OR (Oil, Fish)) OR (Fish Liver Oils)) OR (Liver Oils, Fish)) OR (Oils, Fish Liver)) OR (Fish Oils)
8	(((((((Oils, Fish) OR (Fish Oil)) OR (Oil, Fish)) OR (Fish Liver Oils)) OR (Liver Oils, Fish)) OR (Oils, Fish Liver)) OR (Fish Oils)) AND (((((Cognitions) OR (Cognitive Function)) OR (Cognitive Functions)) OR (Function, Cognitive)) OR (Functions, Cognitive))
9	"Fatty Acids, Omega-6"[Mesh]
10	((((((((((((((Acids, Omega-6 Fatty) OR (Fatty Acids, Omega 6)) OR (N-6 Fatty Acids)) OR (Acids, N-6 Fatty)) OR (Fatty Acids, N-6)) OR (N 6 Fatty Acids)) OR (Omega-6 Fatty Acids)) OR (Omega 6 Fatty Acids)) OR (N-6 Fatty Acid)) OR (Acid, N-6 Fatty)) OR (Fatty Acid, N-6)) OR (N 6 Fatty Acid)) OR (Omega-6 Fatty Acid)) OR (Acid, Omega-6 Fatty)) OR (Fatty Acid, Omega-6)) OR (Omega 6 Fatty Acid)
11	"Fatty Acids, Omega-3"[Mesh]
12	((((((((((((((((((((((((((((N-3 Fatty Acid) OR (Acid, N-3 Fatty)) OR (Fatty Acid, N-3)) OR (N 3 Fatty Acid)) OR (Omega-3 Fatty Acid)) OR (Acid, Omega-3 Fatty)) OR (Fatty Acid, Omega-3)) OR (Omega 3 Fatty Acid)) OR (Omega-3 Fatty Acids)) OR (n-3 Oil)) OR (n 3 Oil)) OR (Oil, n-3)) OR (n3 Oil)) OR (Oil, n3)) OR (n-3 Fatty Acids)) OR (n 3 Fatty Acids)) OR (n-3 Polyunsaturated Fatty Acid)) OR (n 3 Polyunsaturated Fatty Acid)) OR (n-3 PUFA)) OR (n 3 PUFA)) OR (PUFA, n-3)) OR (n3 Fatty Acid)) OR (Fatty Acid, n3)) OR (n3 PUFA)) OR (PUFA, n3)) OR (n3

	Polyunsaturated Fatty Acid)) OR (n3 Oils)) OR (Omega 3 Fatty Acids)) OR (n-3 Oils)) OR (n 3 Oils)
13	((((((((((((((((((((((((((((((N-3 Fatty Acid) OR (Acid, N-3 Fatty)) OR (Fatty Acid, N-3)) OR (N 3 Fatty Acid)) OR (Omega-3 Fatty Acid)) OR (Acid, Omega-3 Fatty)) OR (Fatty Acid, Omega-3)) OR (Omega 3 Fatty Acid)) OR (Omega-3 Fatty Acids)) OR (n-3 Oil)) OR (n 3 Oil)) OR (Oil, n-3)) OR (n3 Oil)) OR (Oil, n3)) OR (n-3 Fatty Acids)) OR (n 3 Fatty Acids)) OR (n-3 Polyunsaturated Fatty Acid)) OR (n 3 Polyunsaturated Fatty Acid)) OR (n-3 PUFA)) OR (n 3 PUFA)) OR (PUFA, n-3)) OR (n3 Fatty Acid)) OR (Fatty Acid, n3)) OR (n3 PUFA)) OR (PUFA, n3)) OR (n3 Polyunsaturated Fatty Acid)) OR (n3 Oils)) OR (Omega 3 Fatty Acids)) OR (n-3 Oils)) OR (n 3 Oils)) AND ((((((((((((((((Acids, Omega-6 Fatty) OR (Fatty Acids, Omega 6)) OR (N-6 Fatty Acids)) OR (Acids, N-6 Fatty)) OR (Fatty Acids, N-6)) OR (N 6 Fatty Acids)) OR (Omega-6 Fatty Acids)) OR (Omega 6 Fatty Acids)) OR (N-6 Fatty Acid)) OR (Acid, N-6 Fatty)) OR (Fatty Acid, N-6)) OR (N 6 Fatty Acid)) OR (Omega-6 Fatty Acid)) OR (Acid, Omega-6 Fatty)) OR (Fatty Acid, Omega-6)) OR (Omega 6 Fatty Acid))
14	(((((((((((((((((((((((((((((((N-3 Fatty Acid) OR (Acid, N-3 Fatty)) OR (Fatty Acid, N-3)) OR (N 3 Fatty Acid)) OR (Omega-3 Fatty Acid)) OR (Acid, Omega-3 Fatty)) OR (Fatty Acid, Omega-3)) OR (Omega 3 Fatty Acid)) OR (Omega-3 Fatty Acids)) OR (n-3 Oil)) OR (n 3 Oil)) OR (Oil, n-3)) OR (n3 Oil)) OR (Oil, n3)) OR (n-3 Fatty Acids)) OR (n 3 Fatty Acids)) OR (n-3 Polyunsaturated Fatty Acid)) OR (n 3 Polyunsaturated Fatty Acid)) OR (n-3 PUFA)) OR (n 3 PUFA)) OR (PUFA, n-3)) OR (n3 Fatty Acid)) OR (Fatty Acid, n3)) OR (n3 PUFA)) OR (PUFA, n3)) OR (n3 Polyunsaturated Fatty Acid)) OR (n3 Oils)) OR (Omega 3 Fatty Acids)) OR (n-3 Oils)) OR (n 3 Oils)) AND ((((((((((((((((Acids, Omega-6 Fatty) OR (Fatty Acids, Omega 6)) OR (N-6 Fatty Acids)) OR (Acids, N-6 Fatty)) OR (Fatty Acids, N-6)) OR (N 6 Fatty Acids)) OR (Omega-6 Fatty Acids)) OR (Omega 6 Fatty Acids)) OR (N-6 Fatty Acid)) OR (Acid, N-6 Fatty)) OR (Fatty Acid, N-6)) OR (N 6 Fatty Acid)) OR (Omega-6 Fatty Acid)) OR (Acid, Omega-6 Fatty)) OR (Fatty Acid, Omega-6)) OR (Omega 6 Fatty Acid))) AND (((((Cognitions) OR (Cognitive Function)) OR (Cognitive Functions)) OR (Function, Cognitive)) OR (Functions, Cognitive))
15	"Docosahexaenoic Acids"[Mesh]
16	(((((((((((((((((Acids, Docosahexaenoic) OR (Docosahexenoic Acids)) OR (Acids, Docosahexenoic)) OR (Docosahexaenoic Acid)) OR (Acid, Docosahexaenoic)) OR (Docosahexaenoic Acid (All-Z Isomer))) OR (Docosahexaenoic Acid, 4,7,10,13,16,19-(All-Z-Isomer))) OR (Docosahexaenoic Acid, 4,7,10,13,16,19-Isomer, Sodium Salt)) OR (Docosahexaenoic Acid, 3,6,9,12,15,18-Isomer)) OR (Docosahexaenoic Acid, Sodium Salt)) OR (Docosahexaenoic Acid, 4,7,10,13,16,19-Isomer)) OR (Docosahexaenoic Acid, 4,7,10,13,16,19-(All-Z-Isomer), Potassium Salt)) OR (Docosahexaenoic Acid Dimer (All-Z Isomer))) OR (Docosahexaenoic Acid, 4,7,10,13,16,19-(All-Z-Isomer), Cesium Salt)) OR (Docosahexaenoic Acid, 4,7,10,13,16,19-(All-Z-Isomer), Cerium Salt)) OR (Docosahexaenoate)) OR (Docosahexaenoic Acid, 4,7,10,13,16,19-(Z,Z,Z,Z,Z,E-Isomer))

17	"Arachidonic Acid"[Mesh]
18	((((((((((((((all-Z)-5,8,11,14-Eicosatetraenoic acid) OR (Vitamin F)) OR (Arachidonic Acid, Sodium Salt)) OR (Sodium Arachidonate)) OR (Arachidonate, Sodium)) OR (Arachidonic Acid, Cesium Salt, (all-Z)-Isomer)) OR (Arachidonic Acid, Cerium Salt, (all-Z)-Isomer)) OR (Arachidonic Acid, Potassium Salt, (all-Z)-Isomer)) OR (Arachidonic Acid, (all-Z)-Isomer, 1-(14)C-Labeled)) OR (Arachidonic Acid, Ammonium Salt, (all-Z)-Isomer)) OR (Arachidonic Acid, Zinc Salt, (all-Z)-Isomer)) OR (Arachidonic Acid, Sodium Salt, (all-Z)-Isomer)) OR (Arachidonic Acid, (all-Z)-isomer, 3H-Labeled)) OR (Arachidonic Acid, Lithium Salt, (all-Z)-Isomer)
19	((((((((((((((all-Z)-5,8,11,14-Eicosatetraenoic acid) OR (Vitamin F)) OR (Arachidonic Acid, Sodium Salt)) OR (Sodium Arachidonate)) OR (Arachidonate, Sodium)) OR (Arachidonic Acid, Cesium Salt, (all-Z)-Isomer)) OR (Arachidonic Acid, Cerium Salt, (all-Z)-Isomer)) OR (Arachidonic Acid, Potassium Salt, (all-Z)-Isomer)) OR (Arachidonic Acid, (all-Z)-Isomer, 1-(14)C-Labeled)) OR (Arachidonic Acid, Ammonium Salt, (all-Z)-Isomer)) OR (Arachidonic Acid, Zinc Salt, (all-Z)-Isomer)) OR (Arachidonic Acid, Sodium Salt, (all-Z)-Isomer)) OR (Arachidonic Acid, (all-Z)-isomer, 3H-Labeled)) OR (Arachidonic Acid, Lithium Salt, (all-Z)-Isomer)) AND ((((((((((((((((Acids, Docosahexaenoic) OR (Docosahexenoic Acids)) OR (Acids, Docosahexenoic)) OR (Docosahexaenoic Acid)) OR (Acid, Docosahexaenoic)) OR (Docosahexaenoic Acid (All-Z Isomer))) OR (Docosahexaenoic Acid, 4,7,10,13,16,19-(All-Z-Isomer))) OR (Docosahexaenoic Acid, 4,7,10,13,16,19-Isomer, Sodium Salt)) OR (Docosahexaenoic Acid, 3,6,9,12,15,18-Isomer)) OR (Docosahexaenoic Acid, Sodium Salt)) OR (Docosahexaenoic Acid, 4,7,10,13,16,19-Isomer)) OR (Docosahexaenoic Acid, 4,7,10,13,16,19-(All-Z-Isomer), Potassium Salt)) OR (Docosahexaenoic Acid Dimer (All-Z Isomer))) OR (Docosahexaenoic Acid, 4,7,10,13,16,19-(All-Z-Isomer), Cesium Salt)) OR (Docosahexaenoic Acid, 4,7,10,13,16,19-(All-Z-Isomer), Cerium Salt)) OR (Docosahexaenoate)) OR (Docosahexaenoic Acid, 4,7,10,13,16,19-(Z,Z,Z,Z,Z,E-Isomer)))
20	((((((((((((((all-Z)-5,8,11,14-Eicosatetraenoic acid) OR (Vitamin F)) OR (Arachidonic Acid, Sodium Salt)) OR (Sodium Arachidonate)) OR (Arachidonate, Sodium)) OR (Arachidonic Acid, Cesium Salt, (all-Z)-Isomer)) OR (Arachidonic Acid, Cerium Salt, (all-Z)-Isomer)) OR (Arachidonic Acid, Potassium Salt, (all-Z)-Isomer)) OR (Arachidonic Acid, (all-Z)-Isomer, 1-(14)C-Labeled)) OR (Arachidonic Acid, Ammonium Salt, (all-Z)-Isomer)) OR (Arachidonic Acid, Zinc Salt, (all-Z)-Isomer)) OR (Arachidonic Acid, Sodium Salt, (all-Z)-Isomer)) OR (Arachidonic Acid, (all-Z)-isomer, 3H-Labeled)) OR (Arachidonic Acid, Lithium Salt, (all-Z)-Isomer)) AND ((((((((((((((((Acids, Docosahexaenoic) OR (Docosahexenoic Acids)) OR (Acids, Docosahexenoic)) OR (Docosahexaenoic Acid)) OR (Acid, Docosahexaenoic)) OR (Docosahexaenoic Acid (All-Z Isomer))) OR (Docosahexaenoic Acid, 4,7,10,13,16,19-(All-Z-Isomer))) OR (Docosahexaenoic Acid, 4,7,10,13,16,19-Isomer, Sodium Salt)) OR (Docosahexaenoic Acid, 3,6,9,12,15,18-Isomer)) OR (Docosahexaenoic Acid, Sodium Salt)) OR (Docosahexaenoic Acid, 4,7,10,13,16,19-Isomer)) OR (Docosahexaenoic Acid,

	4,7,10,13,16,19-(All-Z-Isomer), Potassium Salt)) OR (Docosahexaenoic Acid Dimer (All-Z Isomer))) OR (Docosahexaenoic Acid, 4,7,10,13,16,19-(All-Z-Isomer), Cesium Salt)) OR (Docosahexaenoic Acid, 4,7,10,13,16,19-(All-Z-Isomer), Cerium Salt)) OR (Docosahexaenoate)) OR (Docosahexaenoic Acid, 4,7,10,13,16,19-(Z,Z,Z,Z,Z,E-Isomer)))) AND (((((Cognitions) OR (Cognitive Function)) OR (Cognitive Functions)) OR (Function, Cognitive)) OR (Functions, Cognitive))

Table S2. Literature search strategy in web of science.

1	Cognition (Topic) OR Cognitions (Topic) OR Cognitive Function (Topic) OR Cognitive Functions (Topic) OR Function, Cognitive (Topic) OR Functions, Cognitive (Topic) and Preprint Citation Index (Exclude – Database)
2	Fatty Acids (Topic) OR Fatty Acid (Topic) OR Aliphatic Acids (Topic) OR Aliphatic Acid (Topic) OR Acid, Aliphatic (Topic) OR Fatty Acids, Esterified (Topic) OR Esterified Fatty Acids (Topic) OR Esterified Fatty Acid (Topic) OR Acid, Esterified Fatty (Topic) OR Fatty Acid, Esterified (Topic) OR Fatty Acids, Saturated (Topic) OR Saturated Fatty Acids (Topic) OR Saturated Fatty Acid (Topic) OR Acid, Saturated Fatty (Topic) OR Fatty Acid, Saturated (Topic) and Preprint Citation Index (Exclude – Database)
3	#2 AND #1 and Preprint Citation Index (Exclude – Database)
4	Fish Oils (Topic) OR Oils, Fish (Topic) OR Fish Oil (Topic) OR Oil, Fish (Topic) OR Fish Liver Oils (Topic) OR Liver Oils, Fish (Topic) OR Oils, Fish Liver (Topic) and Preprint Citation Index (Exclude – Database)
5	#4 AND #1 and Preprint Citation Index (Exclude – Database)
6	Fatty Acids, Omega-6 (Topic) OR Acids, Omega-6 Fatty (Topic) OR Fatty Acids, Omega 6 (Topic) OR N-6 Fatty Acids (Topic) OR Acids, N-6 Fatty (Topic) OR Fatty Acids, N-6 (Topic) OR N 6 Fatty Acids (Topic) OR Omega-6 Fatty Acids (Topic) OR Omega 6 Fatty Acids (Topic) OR N-6 Fatty Acid (Topic) OR Acid, N-6 Fatty (Topic) OR Fatty Acid, N-6 (Topic) OR N 6 Fatty Acid (Topic) OR Omega-6 Fatty Acid (Topic) OR Acid, Omega-6 Fatty (Topic) OR Fatty Acid, Omega-6 (Topic) OR Omega 6 Fatty Acid (Topic) and Preprint Citation Index (Exclude – Database)
7	Fatty Acids, Omega-3 (Topic) OR N-3 Fatty Acid (Topic) OR Acid, N-3 Fatty (Topic) OR Fatty Acid, N-3 (Topic) OR N 3 Fatty Acid (Topic) OR Omega-3 Fatty Acid (Topic) OR Acid, Omega-3 Fatty (Topic) OR Fatty Acid, Omega-3 (Topic) OR Omega 3 Fatty Acid (Topic) OR Omega-3 Fatty Acids (Topic) OR n-3 Oil (Topic) OR n 3 Oil (Topic) OR Oil, n-3 (Topic) OR n3 Oil (Topic) OR Oil, n3 (Topic) OR n-3 Fatty Acids (Topic) OR n 3 Fatty Acids (Topic) AND n-3 Polyunsaturated Fatty Acid (Topic) AND n 3 Polyunsaturated Fatty Acid (Topic) AND n-3 PUFA (Topic) AND n 3 PUFA (Topic) AND PUFA, n-3 (Topic) OR n3 Fatty Acid (Topic) AND Fatty Acid, n3 (Topic) AND n3 PUFA (Topic) AND PUFA, n3 (Topic) AND n3 Polyunsaturated Fatty Acid (Topic) AND n3 Oils (Topic) AND Omega 3 Fatty Acids (Topic) AND n-3 Oils (Topic) AND n 3 Oils (Topic) and Preprint Citation Index (Exclude – Database)
8	#7 AND #6 and Preprint Citation Index (Exclude – Database)
9	#8 AND #1 and Preprint Citation Index (Exclude – Database)
10	Docosahexaenoic Acids (Topic) OR Acids, Docosahexaenoic (Topic) OR Docosahexenoic Acids (Topic) OR Acids, Docosahexenoic (Topic) OR

	Docosahexaenoic Acid (Topic) OR Acid, Docosahexaenoic (Topic) OR Docosahexaenoic Acid (All-Z Isomer) (Topic) OR Docosahexaenoic Acid, 4,7,10,13,16,19-(All-Z-Isomer) (Topic) OR Docosahexaenoic Acid, 4,7,10,13,16,19-Isomer, Sodium Salt (Topic) OR Docosahexaenoic Acid, 3,6,9,12,15,18-Isomer (Topic) OR Docosahexaenoic Acid, Sodium Salt (Topic) OR Docosahexaenoic Acid, 4,7,10,13,16,19-Isomer (Topic) OR Docosahexaenoic Acid, 4,7,10,13,16,19-(All-Z-Isomer), Potassium Salt (Topic) OR Docosahexaenoic Acid Dimer (All-Z Isomer) (Topic) OR Docosahexaenoic Acid, 4,7,10,13,16,19-(All-Z-Isomer), Cesium Salt (Topic) OR Docosahexaenoic Acid, 4,7,10,13,16,19-(All-Z-Isomer), Cerium Salt (Topic) OR Docosahexaenoate (Topic) OR Docosahexaenoic Acid, 4,7,10,13,16,19-(Z,Z,Z,Z,Z,E-Isomer) (Topic) and Preprint Citation Index (Exclude – Database)
11	Arachidonic Acid (Topic) OR (all-Z)-5,8,11,14-Eicosatetraenoic acid (Topic) OR Vitamin F (Topic) OR Arachidonic Acid, Sodium Salt (Topic) OR Sodium Arachidonate (Topic) OR Arachidonate, Sodium (Topic) OR Arachidonic Acid, Cesium Salt, (all-Z)-Isomer (Topic) OR Arachidonic Acid, Cerium Salt, (all-Z)-Isomer (Topic) OR Arachidonic Acid, Potassium Salt, (all-Z)-Isomer (Topic) OR Arachidonic Acid, (all-Z)-Isomer, 1-(14)C-Labeled (Topic) OR Arachidonic Acid, Ammonium Salt, (all-Z)-Isomer (Topic) OR Arachidonic Acid, Zinc Salt, (all-Z)-Isomer (Topic) OR Arachidonic Acid, Sodium Salt, (all-Z)-Isomer (Topic) OR Arachidonic Acid, (all-Z)-isomer, 3H-Labeled (Topic) OR Arachidonic Acid, Lithium Salt, (all-Z)-Isomer (Topic) and Preprint Citation Index (Exclude – Database)
12	#11 AND #10 and Preprint Citation Index (Exclude – Database)
13	#12 AND #1 and Preprint Citation Index (Exclude – Database)

Table S3. Literature search strategy in EMBASE

1	cognition OR cognitions OR (cognitive AND function) OR (cognitive AND functions) OR (function, AND cognitive) OR (functions, AND cognitive)
2	fatty AND acids OR (fatty AND acid) OR (aliphatic AND acids) OR (aliphatic AND acid) OR (acid, AND aliphatic) OR (fatty AND acids, AND esterified) OR (esterified fatty AND acids) OR (esterified fatty AND acid) OR (acid, AND esterified AND fatty) OR (fatty AND acid, AND esterified) OR (fatty AND acids, AND saturated) OR (saturated fatty AND acids) OR (saturated fatty AND acid) OR (acid, AND saturated AND fatty) OR (fatty AND acid, AND saturated)
3	#1 AND #2
4	fish AND oils OR (oils, AND fish) OR (fish AND oil) OR (oil, AND fish) OR (fish AND liver AND oils) OR (liver AND oils, AND fish) OR (oils, AND fish AND liver)
5	#1 AND #4
6	fatty AND 'acids, omega 6' OR 'acids, omega 6 fatty' OR (fatty AND acids, omega AND 6) OR ('n 6' AND fatty AND acids) OR (acids, AND 'n 6' AND fatty) OR (fatty AND acids, AND 'n 6') OR (n AND 6 AND fatty AND acids) OR ('omega 6 fatty' AND acids) OR (omega AND 6 fatty AND acids) OR ('n 6' AND fatty AND acid) OR (acid, AND 'n 6' AND fatty) OR (fatty AND acid, AND 'n 6') OR (n AND 6 AND fatty AND acid) OR ('omega 6 fatty' AND acid) OR 'acid, omega 6 fatty' OR (fatty AND 'acid, omega 6') OR (omega AND 6 fatty AND acid)
7	fatty AND acids, AND 'omega 3' OR ('n 3' AND fatty AND acid) OR (acid, AND 'n 3' AND fatty) OR (fatty AND acid, AND 'n 3') OR (n AND 3 AND fatty AND acid) OR ('omega 3 fatty' AND acid) OR 'acid, omega 3 fatty' OR (fatty AND 'acid, omega 3') OR (omega AND 3 fatty AND acid) OR ('omega 3 fatty' AND acids) OR ('n 3' AND oil) OR (n AND 3 AND oil) OR (oil, AND 'n 3') OR (n3 AND oil) OR (oil, AND n3) OR ('n 3' AND fatty AND acids) OR (omega AND 6 fatty AND acid) OR (n AND 3 AND fatty AND acids) OR ('n 3' AND polyunsaturated AND fatty AND acid) OR (n AND 3 AND polyunsaturated AND fatty AND acid) OR ('n 3' AND pufa) OR (n AND 3 AND pufa) OR (pufa, AND 'n 3') OR (n3 AND fatty AND acid) OR (fatty AND acid, AND n3) OR (n3 AND pufa) OR (pufa, AND n3) OR (n3 AND polyunsaturated AND fatty AND acid) OR (n3 AND oils) OR (omega AND 3 fatty AND acids) OR ('n 3' AND oils) OR (n AND 3 AND oils)
8	#6 AND #7
9	#1 AND #8
10	dhasco OR docosahexaenoate OR (docosahexaenoic AND acids) OR (docosahexenoic AND acid) OR (docosahexaenoic AND acid)
11	5, AND 8, AND 11, AND 14 AND eicosatetraenoic AND acid OR (all AND cis AND 5, AND 8, AND 11, AND 14 AND eicosatetraenoic AND acid) OR arachidonate OR (arachidonate AND sodium) OR (delta5, AND 8, AND 11, AND 14 AND eicosatetraenoic AND acid) OR (eicosa AND 5, AND 8, AND 11, AND 14 AND tetraenoic AND acid) OR (sodium AND arachidonate) OR (arachidonic AND acid)
12	#10 AND #11
13	#1 AND #12

(A) Sensitivity analysis of cognitive function.

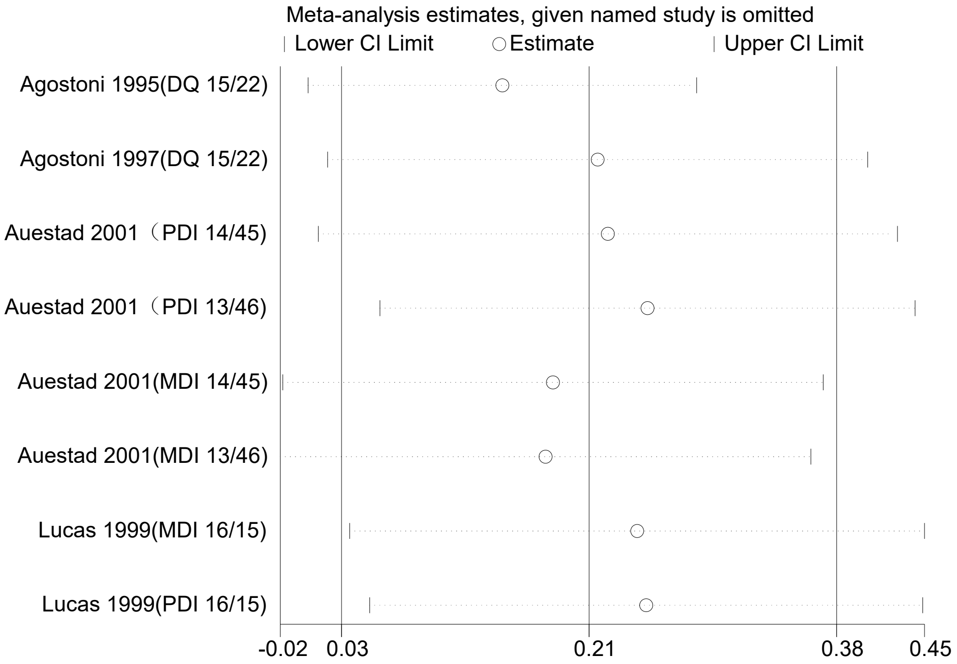

(B) Sensitivity analysis of PDI.

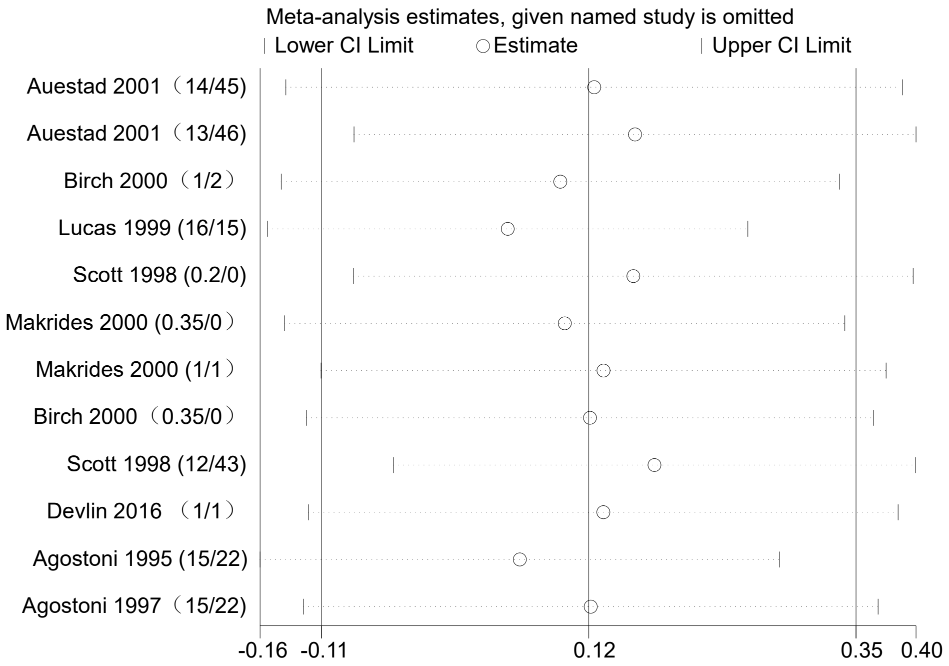

(C) Sensitivity analysis of MDI.

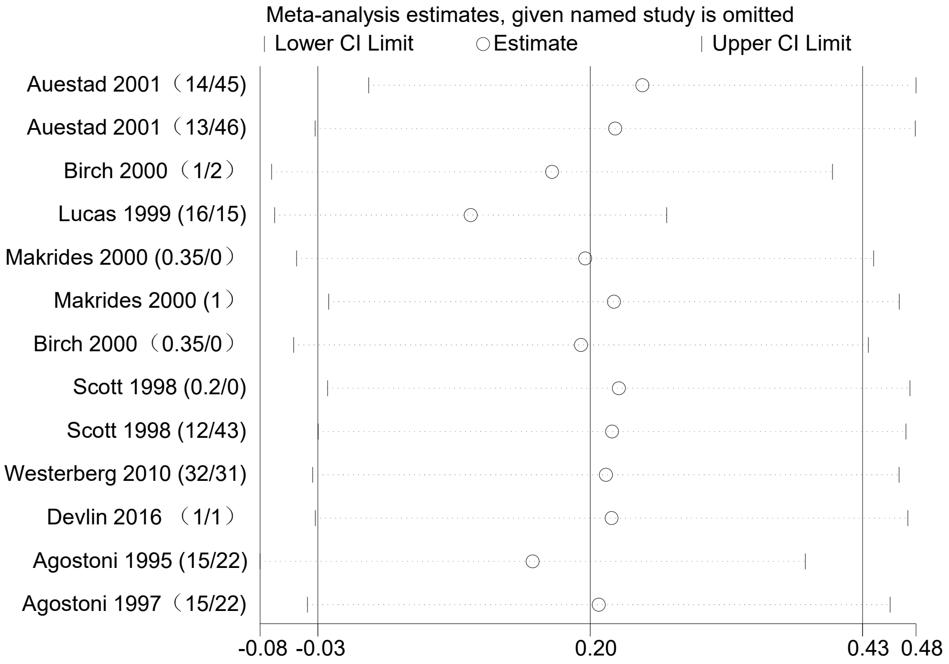

Figure S1. Sensitivity analysis of the effects of DHA and ARA supplementation on cognition development.

(A)Funnel plot for assessing publication bias on cognition development.

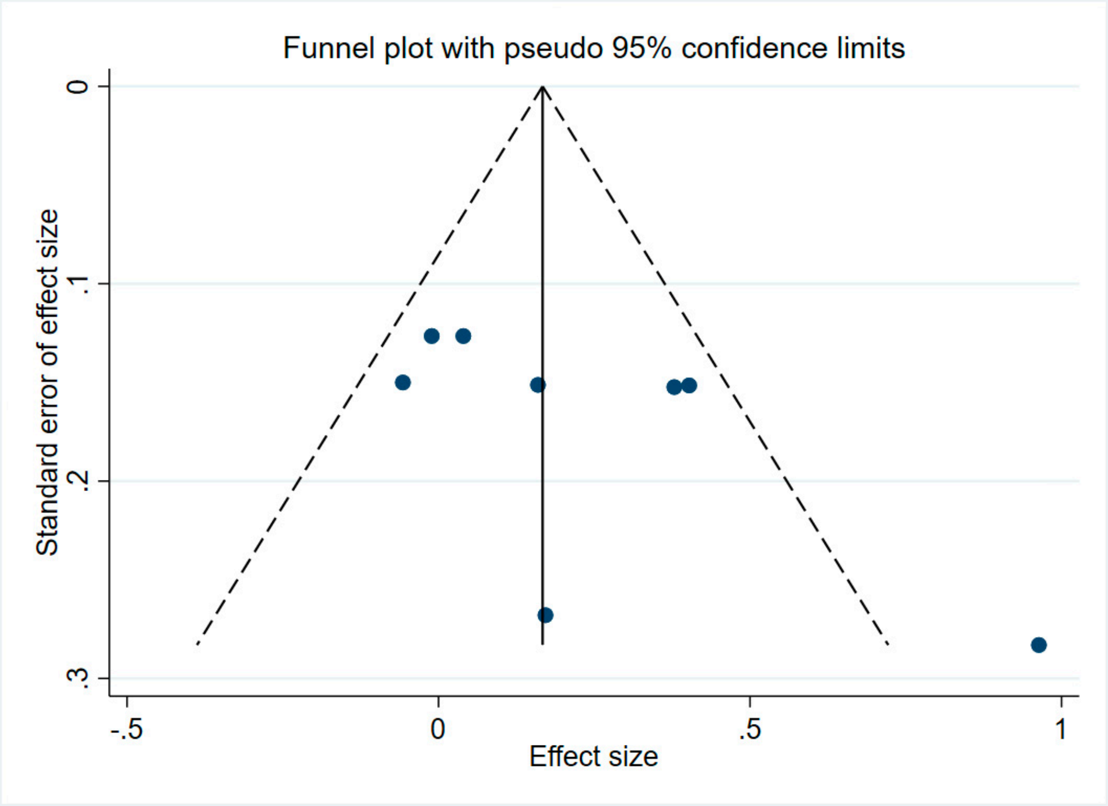

(B) Funnel plot for assessing publication bias on PDI.

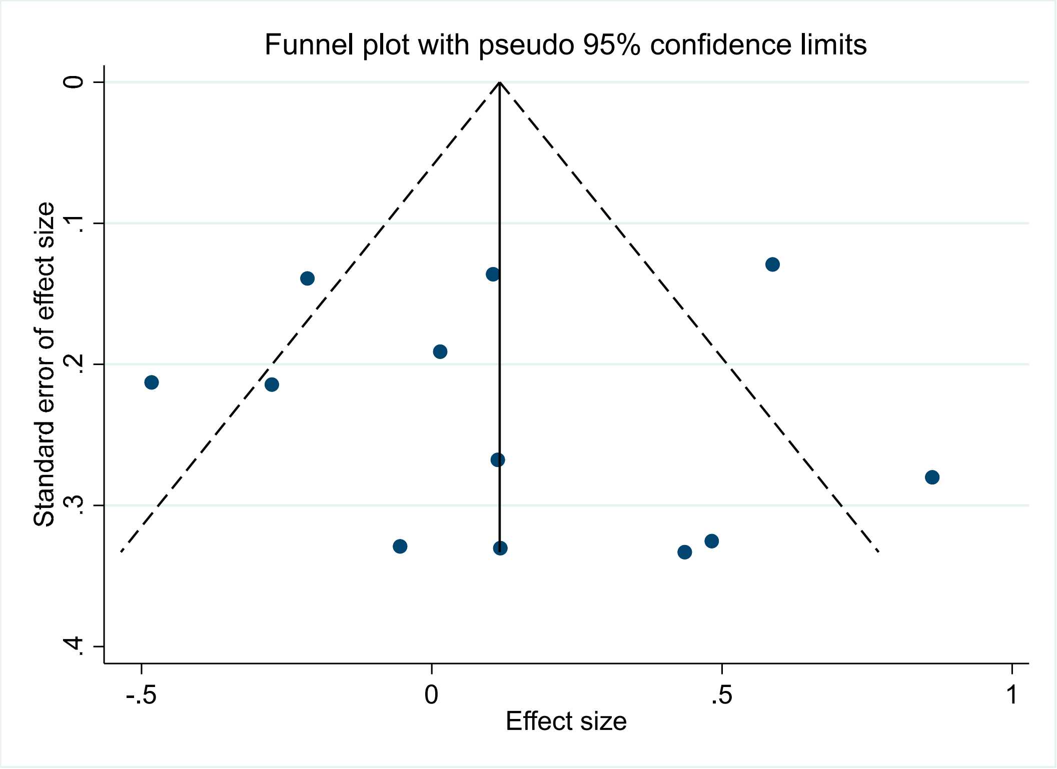

(C)Funnel plot for assessing publication bias on MDI.

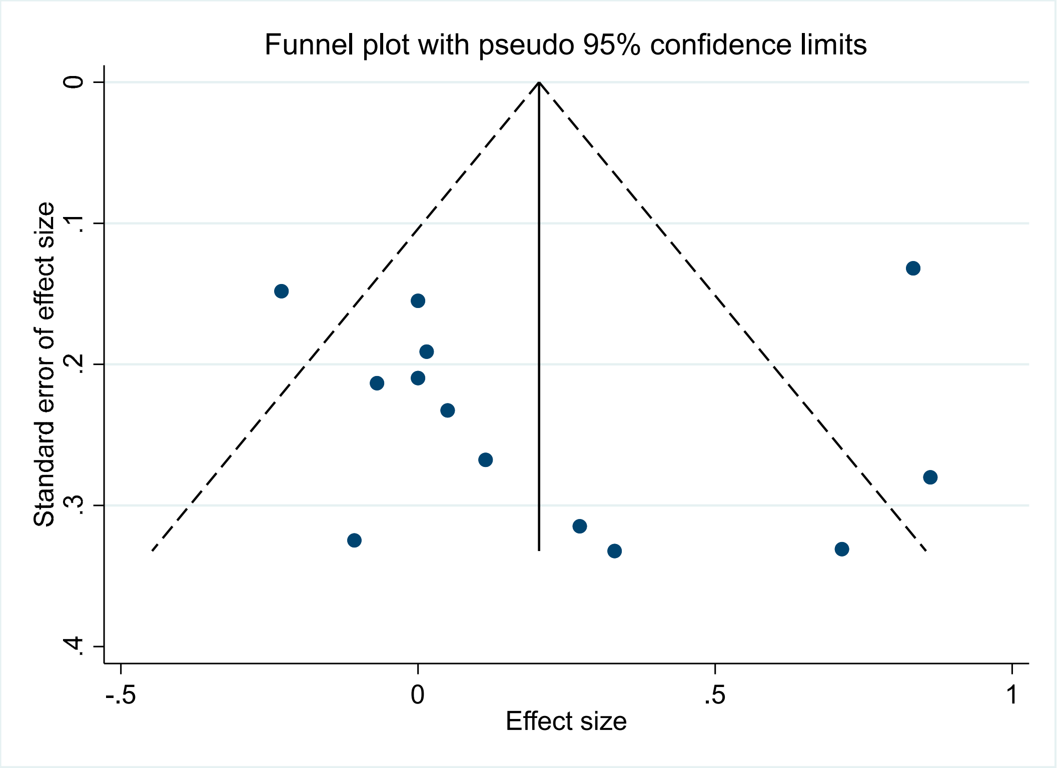

Figure S2. Funnel plot for assessing publication bias in the studies reporting the effect of DHA and ARA intake on PDI and MDI.
